# Effects of the COVID-19 Pandemic on Parkinson’s Disease: a Single-Centered Qualitative Study

**DOI:** 10.1017/cjn.2021.70

**Published:** 2021-04-12

**Authors:** Beatrice Ana-Maria Anghelescu, Veronica Bruno, Davide Martino, Pamela Roach

**Affiliations:** Department of Clinical Neurosciences, University of Calgary, Calgary, Canada; Hotchkiss Brain Institute, University of Calgary, Calgary, Canada; O’Brien Institute of Public Health, University of Calgary, Calgary, Canada; Department of Community Health Sciences, University of Calgary, Calgary, Canada; Department of Family Medicine, University of Calgary, Calgary, Canada

**Keywords:** COVID-19, Parkinson’s disease, Pandemic, Quality of life, Quality of care, Qualitative

## Abstract

**Background:**

The public health measure restrictions across the world due to COVID-19 have inadvertently impacted the routines for people with Parkinson’s disease (PD) and their care partners not only in terms of compromised neurological clinical care but also drastically changing the way of life to minimize the risk of becoming infected. This study explores initial PD patients’ lived experiences to observe how quality of life and health care has been affected at the start of the COVID-19 pandemic and provide insight into the importance of patient engagement and virtual care.

**Methods:**

Twenty-two virtual, in-depth semi-structured interviews with persons diagnosed with PD who usually attend a Movement Disorders specialty clinic in Calgary, Alberta, were completed between April 28 and May 13, 2020, and the care partners that wished to participate. Interviews were recorded and transcribed, after which transcripts were analyzed and coded into relevant themes using NVivo 12.

**Results:**

Impacts from the public health measures and COVID-19 results into three main themes: (1) Impacts of COVID-19 on PD Clinical Care; (2) Activities of Daily Living; (3) Attitudes and Perceptions. Participants reported worsening in motor and nonmotor symptoms and had to accommodate to clinical care via virtual means which were associated with limitations and suggestions for improvement of remote care.

**Conclusion:**

This study provides a unique opportunity for researchers to better understand the lived experiences of PD patients in all aspects of their life suggesting that innovative means are needed for facilitating virtual health care medicine and increased social interaction.

## RÉSUMÉ

Effets de la pandémie de COVID-19 sur des patients atteints de la maladie de Parkinson : une étude menée dans un établissement de santé au Canada.

**Contexte:**

Les restrictions relatives à la santé publique imposées partout dans le monde en raison de la pandémie de COVID-19 ont eu un impact involontaire sur la routine des patients atteints de la maladie de Parkinson (MP) et sur leurs prestataires de soins, d’abord en ce qui concerne les soins neurologiques offerts mais aussi en ce qui regarde les habitudes de vie des patients, lesquelles ont été modifiées de manière draconienne pour éviter les risques d’infection. Cette étude entend donc se pencher sur l’expérience initiale des patients atteints de la MP et observer de quelle façon leur qualité de vie et les soins qui leur sont prodigués ont été affectés au début de la pandémie de COVID-19. Elle souhaite aussi donner un aperçu de l'importance de l'engagement des patients et des soins virtuels.

**Méthodes:**

Vingt-deux entretiens approfondis semi-structurés ont été effectués virtuellement du 28 avril au 13 mai 2020 avec des individus chez qui l’on avait diagnostiqué la MP et qui fréquentaient habituellement une clinique spécialisée des troubles du mouvement située à Calgary (Alberta) ainsi qu’avec des prestataires de soins qui souhaitaient y participer. Ces entretiens ont été tout d’abord enregistrés ; on a ensuite analysé et codé leurs transcriptions en fonction de thèmes pertinents en utilisant NVivo 12.

**Résultats:**

Les impacts des restrictions relatives à la santé publique ont pu être catégorisés en fonction de trois thèmes principaux : 1) les impacts de la pandémie de COVID-19 sur les soins cliniques destinés aux patients atteints de la MP ; 2) les activités de la vie quotidienne ; 3) les attitudes et les perceptions. De façon générale, les participants ont signalé une aggravation de leurs symptômes moteurs et non moteurs et ont dû composer avec des soins cliniques offerts par des moyens virtuels. Ces soins et ces moyens ont été par ailleurs associés à certaines limites et à des suggestions pour améliorer l’offre de service à distance.

**Conclusion:**

Cette étude offre donc aux chercheurs une possibilité unique de mieux comprendre tous les aspects de l’expérience des patients atteints de la MP. Elle suggère que des moyens innovateurs sont nécessaires pour faciliter la médecine virtuelle et accroître les interactions sociales.

## Introduction

With the first reported COVID-19 (SARS-CoV-2) case in December 2019, the World Health Organization declared a pandemic resulting in varying levels of social distancing and public health measure restrictions across the world. To mitigate the spread of COVID-19, personal practices and community-based measures were set in place, including limits on public health gatherings, non-essential businesses, non-urgent clinical care, and travel. Despite the existing safety precautions in place, COVID-19 has increased the vulnerability for certain populations, specifically the elderly, those with comorbidities, and patients living with a chronic disease such as Parkinson’s disease (PD).^1^

These measures inadvertently impacted the routines for people with PD and their care partners in compromised neurological clinical care and drastically changed the way of life to minimize the risk of becoming infected playing a negative role in PD patients.^2^ The rapid progression of the pandemic and governments response to the reorganization of the economy has limited health care resources, and placed PD patients at risk in deterioration of motor-worsening and indirect effects including non-motor negative consequences of stress, self-isolation, anxiety, and depression.^3-5^ The public health restrictions have further impacted the way PD patients obtain medications for their management of the disease.

Overall, the health care sector has undergone rapid and drastic modifications to cope with current needs, with routine clinic visits consequently being on hold to address acute care.^6^ Alberta reported 315 new COVID-19 cases and 7 more COVID-19-related deaths by April 29, 2020, with a total of 3,590 cases in the Calgary zone. The Movement Disorders Clinic at the Foothills Medical Centre Calgary, Alberta, suspended their clinics on March 13, 2020, cancelling all in-person clinical appointments and transitioning to a new growing field of telemedicine. With the second wave of COVID-19 approaching, continued virtual care becomes increasingly critical to understand. A purely qualitative approach on PD experiences with virtual care and COVID-19 is missing. While there is new emerging quantitative studies^7,8^ on PD and COVID-19, a qualitative approach on perceptions, attitudes, and experiences has been limited, leaving patient-centered outcomes to be overshadowed during this time. Our single-center study provides insight into the direct and indirect associations between COVID-19 and PD clinical care and other daily effects, which may serve as guidance in the current and upcoming times of telemedicine.

The present study aimed to understand the impacts of COVID-19 on patients’ clinical symptoms of PD in both motor and non-motor aspects and learn from the direct experiences of virtual medicine from PD patients that could help guide social and health services virtually moving forward. We conducted a qualitative study with patients who receive community-based care at a specialty Movement Disorders clinic. A qualitative approach with a reflexive thematic approach to context analysis was used to collect rich data and develop in-depth understanding on this population’s lived experience to better inform health care management for patients living with PD during a pandemic and utilizing virtual care. This study utilizes patient engagement to drive understanding and next steps for future virtual care.

## Research Design

We completed 22 virtual, in-depth semi-structured interviews with persons diagnosed with PD between April 28 and May 13, 2020, and their care partners if they wished to attend the interviews and were able to provide consent. To provide current and up-to-date data on the pandemic’s impacts, participants were enrolled and provided detail on their experience while the pandemic was occurring, and public health measures were in place.

### Recruitment

We purposely recruited participants who are regularly followed up by the Movement Disorders Clinic in Calgary, Alberta, Canada. Participants had a diagnosis of PD verified by a specialty neurologist according to the UK Brain Bank diagnostic criteria,^9^ had agreed to participate in the CaPRI (Calgary Parkinson Research Initiative) registry, and had previously provided written consent to be contacted about additional research. CaPRI registry’s purpose is to increase our understanding and knowledge of PD or other Parkinsonian syndromes to develop better tests to diagnose and improve the management and treatments of the disease. Consecutive patients from the CaPRI registry who had experiences in telemedicine in the last 6 weeks or had an upcoming virtual appointment were included.

Potential participants were contacted by telephone and informed of the project, after which information sheets and consent forms specific to the study were emailed shortly after to interested participants following local IRB consenting procedures. Participants were given a minimum of 24 h to consider the information and discuss it with family members or health providers before consenting to take part. Participants were given the option of telephone or Zoom interviews for their scheduled interviews.^10^ Zoom is a web-based video conferencing tool that allows a virtual face-to-face meeting. Zoom provided more intimate interaction for those participants longing for the interpersonal connection. It also provides many participants practice with using video conferencing and technology who hoped to be better able to use Zoom amid COVID-19 with friends and family. Alternatively, some participants opted for the convince and simplicity of the telephone interview.

Informed consent was obtained using Qualtrics survey software^11^ in all but one participant who could not connect to the internet to access the consent form and provided explicit oral consent. Despite oral consent only being used by one participant, explicit oral consent was incorporated in this study to ethically include a diverse population who may have encountered other difficulties consenting via Qualtrics. Throughout the study, process consent was used, meaning participants consented once via online consent form and consented again verbally at the scheduled time of the interview to ensure that all participants were still comfortable participating in the study at the time of contact.^12^ In some instances, participants wished to reschedule, and process consent was sought again at the time of the rescheduled contact between researcher and participant. The Conjoint Human Research Ethics Board approved this study at the University of Calgary (REB20-0559).

### Data Collection

In-depth interviews were used in a semi-structured format as they are an appropriate way to gain a rich, full understanding of lived experiences. The semi-structured questionnaire consisted of initial questions relating to the pandemic itself, including normal activities and care before the pandemic. The interview schedule was developed through consultation with clinical experts, academic experts, and patient/family representatives regularly involved in research with the Brain and Mental Health Research Clinics. Further questions captured how daily routines and clinical care have been impacted by adherence to ongoing regulations, exploring physical and mental health strains. Overall attitudes and coping mechanisms were explored, and participants were inquired for their feedback on any improvements in helping them during this social distancing and improving virtual clinical care.

To achieve theoretical saturation the team estimated approximately 15–20 interviews with different participants would likely need to be completed.^13,14^ Therefore, with 22 participants enrolled, the target data quota was reached for allowing diverse and rich coding to be available. Common themes were emerging after approximately 15 completed interviews, with the remaining interviews allowing for member checking to look for confirming or disconfirming experiences, and enhancing rigor. In-depth interviews were recorded with an audio recording device and transcribed immediately after. Field notes were taken during interviews or directly following the interviews and uploaded with the recordings to be reviewed during the analysis. Demographic data obtained were age at the time of participation, gender, and information on whether the participants had been tested or diagnosed for SARS-CoV-2. An initial list of 39 potential participants was obtained from the CaPRI registry list. Twenty-two participants were enrolled; 17 from the original list were contacted but either declined or were unable to be reached. The duration of the interviews ranged from 23m:45s to 1h:05m:44s (mean length = 43m:52s) and were completed either by phone or Zoom depending on participant preference, as previously described. Recruitment and data collection were completed by the main research assistant (BA). The senior investigators oversaw the study process. The interviewer had no previous relationship with any of the participants on the registry list and introduced herself and the reason for work at the initial contact.

**Figure 1.**
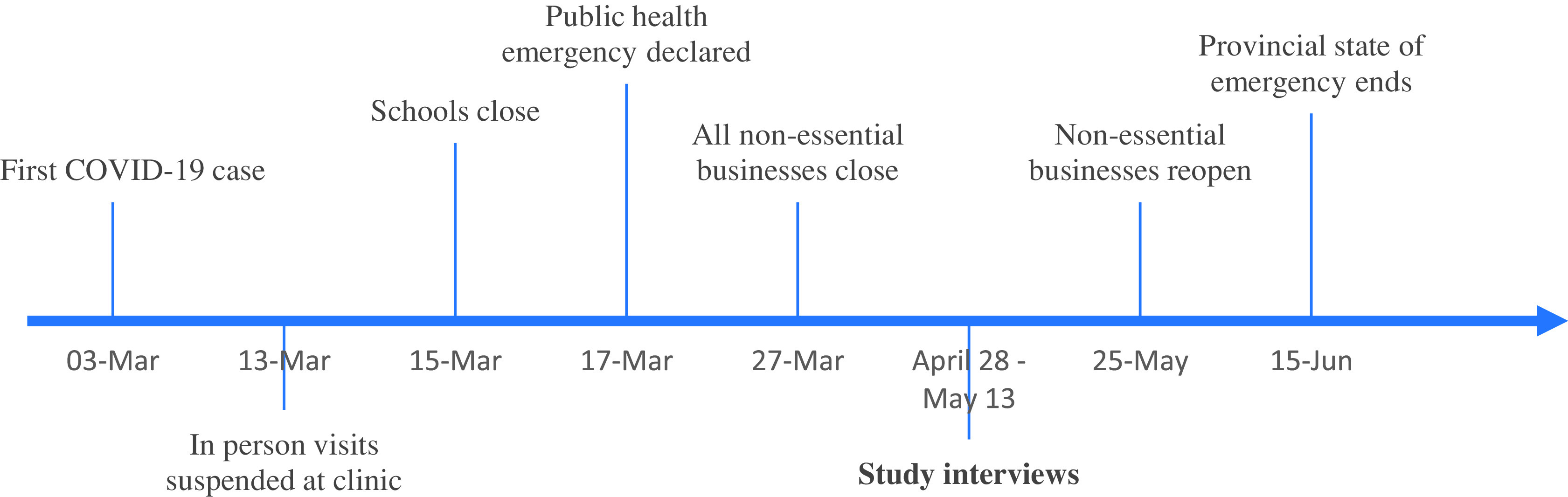
Timeline of COVID-19 Public Health Response in Alberta, Canada.

Figure 1 displays a timeline of the provincial measures for social distancing and health system changes. The Movement Disorders Clinic had stopped in-person clinic visits on March 13, 2020, onwards. Study participants were faced with cancellation of their in-person appointments until further notice, while other appointments were able to be adapted to virtual appointments.

### Analysis

Audio recordings were transcribed verbatim using a secure professional transcribing service and anonymized for any identifying information by the interviewer. Transcripts and field notes were uploaded into NVivo 12 software for qualitative analysis and data management.^15^ To achieve theoretical saturation and generate themes, interviews were transcribed in parallel while data collection was still occurring. The first step of the analysis was to become immersed in the data analysis by having transcripts read and re-read while the interview recordings were replayed. Reflexive thematic analysis was used, which allowed the researchers to be the core in coding and theme development.^13,14^ This form of analysis is well suited for exploring people’s experiences, views, and perceptions. Patterns, analytical thoughts, and iterative analysis were captured through memos and annotations. Research team meetings were held weekly to touch base on emerging codes and themes from the data analysis.

## Results

### Participant Demographics

A total of 22 participants living with a PD diagnosis were included. Five care partners accompanied the PD participants in the interviews and provided additional data. Out of the 22 participants, 16 males contributed 72.7%, and 6 females accounted for 27.3% of our sample. The age range was 51–79 years old with a mean age of 70.5 years of age. No participants reported a diagnosis of COVID-19 for anyone living in the household; two participants received a negative screening test for COVID-19; five participants reported being told by public health authority that they should self-isolate after returning home from travel.

### Thematic Analysis: Experience of the COVID-19 Pandemic

Thematic coding was completed by BA, directed by PR (Principal Investigator and qualitative methods expert) and in collaboration with VB and DM (Movement disorders specialists and researchers).

### Impacts of COVID-19 on PD Clinical Care

#### Healthcare Limitations and Uncertainty

Many patients reported feeling alone and unsupported during the pandemic, expressing limited communication with their physicians and uncertainty around appointments, procedures, and general PD inquiries (Table 1; Subtheme 1.1a Health Care Uncertainty). The public health measures impacted the health services and in turn impacted patients. A few participants stated they had received proper routine care from home care services and reported feeling unsupported when the services stopped (Table 1; Subtheme 1.1b Health Care Uncertainty).

**Table 1.** Theme: impacts of COVID-19 on PD clinical care

Subtheme	Example Quotes
1.1a Health Care Uncertainty	*“The appointment has been cancelled. I don’t have anything virtual on the books. I’m supposed to have the operation, a deep brain stimulation. But I imagine nothing will go ahead until it’s more or less subsided, I guess. I’d like to get on with the deep brain stimulation thing, but I can see where their priorities in the operating room, but it would be handy if I knew what timeframe they’re operating under now.”* (Participant 406)
	*“I couldn’t find it on my calendar because it always reminds me, but I was supposed to have an appointment with her in the next little while, but it’s probably cancelled. I would, I haven’t heard that it was cancelled, or it would be virtual.”* (Participant 411)
1.1b Health Care Limitations	*‘The private care, um continued, except the morning one had to stop because the people were going to see more than one person. Every time they say, they say something ‘Oh, for two weeks you have to do this’ and then they said ‘Oh no, now it’s eight weeks. Now it’s twelve weeks’ Like originally, I was going to lose the services of the shower for a month and then it turned into this twelve weeks, and now its turned into forever, as far as I can tell.”* (Participant 413)
	*“And with Interviewee and with his Parkinson’s, no one, his, um, neurologist, I felt that they could have, maybe should have from their office sent out an email ‘cause they have all our stuff, saying hey just wanted to touch base with what about and maybe this is what you’ve, you know, need to kind of, um, educate yourself with.”* (Participant 421’s spouse)
1.2a Pharmacy and Medication Availability: Dispensing Fees	*“I am annoyed that I can only get 30 days of medicine at a time. So now it’s been a change. So, it’s been annoying to have to get them every 30 days. Well, they have to pay the dispensing fee. If you get three months’ worth for $20 a year, you get one-month worth for $20. 20 jumps to 60 for one medicine.”* (Participant 406)
	*“So cutting it to only one month is- is fine. The doctor gives me three months, and I can say it’s only, it’s only down, uh, the road from here. But first I was thinking it was going to cost me more, but it actually, the- the dispensing fee, if I got it three months’ worth, was one price and if I, um, got just one month at a time, the, with the [insurance], it comes out about the same, so…”* (Participant 411)
	*“Well, it’s, um, taking it every month. My medical plan had to change their limits to what they will pay the pharmacy. So, and they did. Uh, they changed it from five times, um, a, a year to 15, so that, so that they would pay for, um, the prescription every month, uh, and the charges by the pharmacy.”* (Participant, 413)
1.2b Pharmacy and Medication Availability: Routine Change	*“The only problem is … It depends on the week, right? If I wait until I’ve got to go back grocery shopping again, which is every two weeks, I might be able to pick my medications up at the same time and that’s all okay, but if I run out someday before I’ll have to go on another day to the store and other than when I would normally go, but I go once every two weeks normally, if I got to go twice in the same period that’s a little bit of a challenge, but that’s okay, I guess.”* (Participant 400)
	*“No, that, that a, we get our medication, I get my medication through the Pharmacy and, uh, and I just phone them and they send down a month’s worth of my prescriptions. No problem, no, they deliver it to the condo. And it’s not an inconvenience inasmuch as I can go down and pick it up if I, if I want to or I can just have it delivered so it’s not really an inconvenience. No.”* (Participant 419)
1.3a Virtual Medicine: Facilitators	*“Um, so I had another telephone inter, telephone, on Friday, so there’s, there’ve been two, and both were informative and good, and certainly it was impossible for me to get to their office. There was no way I was going to be able to get out of the house, into their offices. So this was good to be able to be in contact with them. Don’t want to go anywhere near (laughs) a doctor’s office. I would pre- much prefer another telephone, um, appointment.”* (Participant 413)
	*“It went fine and actually it works quite well because he was, um, my neurologist was able to, uh, look at me and- and you know, have me do the, the, um, arm motions and so forth which- So sh- the only thing she couldn’t do was sometimes, you know, she actually will take one of my arms and move it around herself, which that wasn’t an option. But everything else, I think, was, worked quite well on Zoom. And, and it, it felt, um, you know, it just was so much easier than, than, you know, going over and trying to find a place to park and waiting in the waiting room and all that. To do it from home took a- actually took away a lot of the stress…of going to an appointment.”* (Participant 417)
1.3b Virtual Medicine: Barriers	*“Um, I think with virtual one of the problems on virtual that you end up speaking at the same time and then somebody stops, you start it, and you stop, and you have to wait and there’s, there’s no, like there’s no management of, uh, when you’re d-, doing, doing face-to-face, you know when a person’s been speaking, and you know you’ve started before they have. But I find that in virtual re-, uh, that virtual kind of stuff, it all becomes quite static. Uh, not static, but staccato. It just is like okay, you start talking. I can’t talk, and then you stop, and I stop, and then who goes first and then, and so I don’t like the, the rhythm to it. Or, uh, I need the body language, I need the eyes, I need, uh, I want to feel, but you don’t feel through the virtual, uh, I want to feel wh-, whether his response is right. Whether he’s hearing me, was he listening to me? I mean, was he looking at me, sort of, of those kinds of things. So, I would never be a fan of the virtual. I would take it over nothing.”* (Participant 415)
	*“So yeah, I thought it was quite well, uh, well. It wasn’t like, uh, video. It was strictly over the phone. And ultimately, uh, you know, I would think, uh, that in person was a better way to go, for a number of reasons. But you know, it worked out pretty good yesterday. Uh, well, just judging on, you know, just recent, uh, I- I wouldn’t say there’s much of an effect. Like I say, I do think it would be better to be in person to have that physical contact- And, uh, the visuals.”* (Participant 405)
	*“I think I can convey all the questions I have. I guess the only disadvantage, well, there’d be two disadvantages. One would be I often put together some material for my neurologist to read, like my current state, what’s the situation with my various symptoms, what I’ve been able to do to overcome them. I like to share that with her. It’s simple. You just put it down on a piece of paper and have her read it rather than me try to articulate. That’s one disadvantage, I guess. But presumably there’ll be a method of sharing it before the Zoom interview.”* (Participant 403)
1.3bc Virtual Medicine: Recommendations	*“One of the things that I’m urging them to do is to set up a secured email service. Because that would give me an ability to let them know right away the impact of some medication changes rather than wait for the next meeting or try to do it through some other mechanism. My family doctor has it set up. So if I have a question for my family doctor, I just have to email on this secured service and they get it to him and he gets back to me. He’ll get back to me and answer my questions and in some cases we haven’t had a need to talk because he’s been able to answer my questions online.* *I think all parts of the health care group should be able to have a secure email service. I’ll bet you it cuts down on the amount of time that doctors spend with patients because you know yourself, if you sit down person to person with somebody, you ask about the weather. You ask about the family. You always kill some time but with email you’re forced to be fairly precise. So it’s a lot faster than having a 15 min appointment.”* (Participant 403)
	*“I think it would be useful if the doctors did use, um, you know, Zoom because then you can look me in the eye to some degree, and look them in the eye.”* (Participant 410)
	*“I think one thing they need to do there is, uh spend money and get a cell phone. Then we could send them you know, face to face. You can just call on our iPhone, just so long as they have, uh, have technology on their phone and then you just put it on, uh face to face, whatever they call it, where we can see each other. It’s just a click of a switch and, and the push of a button and, and you could be seeing each other, just like you were sitting at the table across from each other, and it’s uh, it’s easy.”* (Participant 420)
1.4 Absence of Motor Examination	*“If that’s important to them to track the progress of the symptoms, then it’ll be difficult for them to do that in a virtual method. I never thought it was that useful anyway. Well, it’s probably important to the doctor to use it as a tracking mechanism, but it never really did much for me. I’ve been able to do some things well beyond the normal Parkinson’s stuff. The disadvantage, I suppose, would be from her point of view, she’s not able to… She loves to watch me walk. It’s part of the assessment, so I can’t show her my stuff, that I’m walking probably better than I was in the last visit even. It wouldn’t bother me if we didn’t even do it. It doesn’t do anything for me and I’ve kind of taken care of my motor symptoms with this exercise program, which was endorsed by my neurologist.”* (Participant 403)
	*“You know, it was just pretty much the same as having a, uh, one-on-one visit. The only thing was he couldn’t physically see how I was responding and walking and stuff too well, but other than that, uh, you know, it was certainly adequate. Uh, we have this long enough encounter… close enough relation with our doctors that we kind of understand each other and so it was, it was fine. It was… uh, no issues there, I don’t think. It was, uh, effective, I think.”* (Participant 420)
1.5a Worsening of Motor Symptoms	*“Well, uh, actually, my, uh, my motor symptoms have now I’ve gotten, uh, worse since this, uh, difficult, since the pandemic. Yeah, that’s, uh… My Parkinson’s worse, uh, than it was. I’m worse. I didn’t have that before. In fact, it feels different. I don’t like it, you know, necessarily that. Uh, like I’ve gotten worse and I’m hoping they don’t, uh… I’m hoping when this thing passes, they go back to the way they were.* *Well, I take medication five times a day, and, uh, sometimes between those times I take it, I, I- off times they call it. And the drug didn’t, didn’t affect it, and they are…they are more severe during the off times. They’re often much more now. Make me feel a lot worse than they used to. It varies, uh, a lot. Some days are… Some days or the on day, I really feel good on it. That, that’s rare. But it’s hard to say an average, ‘cause, uh- It varies. You know? But I, I often have two or three hours on the day that, um, I’m not feeling very well.”* (Participant 409)
	*“You know, you have, you have been off a little more. We haven’t really related it to that, uh, but it could be some of the stress. Yeah, it could be anxiety or… we have had to give quite a few extra doses on some nights it’s just that is (laughs) pretty dry, so… we didn’t really think of it that way, but it could be.”* (Participant 414’s spouse)
	*“Um, yeah, this whole thing gets me anxious, and when it does that, like, um, if I’m nervous or something is going on, I tend to shake a little bit more. Um, mainly on my left side. But um, once I get… But the same thing is if I was watching sports, my team’s there, the s-, I get the same reaction is not a- anxious there, but I’m excited, so I… get the- the same movement happens there.* *So, but um, I- I do know in the last, um, it… In the last, um, I guess maybe since this whole virus came about, I do notice that either my medication doesn’t last as long or I’m getting worse, ‘cause the, you don’t know the disease itself how it works, so… I- I still haven’t changed… I’ve changed timing of it a little bit, but I haven’t really taken any more, ‘cause I was set to have, um, the levodopa four times, four times a day… two halves two times a day. But for the longest time, I was only taking one at each of those times. They gave me long, uh, long, reoccurring ones as well. So that was spaced out, so I wasn’t even using the- the eight full tabs a day, so. But now I’m doing it a little more often.” (*Participant 411)
1.5b Worsening of Non-Motor Symptoms	ANXIETY AND STRESS
	*“I have a general, I have a general angst about the, about the COVID19 in that I’m 76 and therefore in the elderly bracket, which, and I’ve got diabetes so uh, that puts me at a greater risk and I’m I have a general anxiety about that.”* (Participant 402)
	*“Yeah. I don’t know whether it’s Parkinson’s related or not, but I’m more anxious because of the things going on the pandemic and the influence that’s had on the economy of our country and province.”* (Participant 403)
	*“I actually did find I lost weight for a while there. I think it was probably stress-related. Now that it seems to be settled into a new routine, I think there’s still some stress, but it’s less stressful than it was.”* (Participant 401)
	MOOD
	*“Um, it’s interesting, I don’t s-, really feel any more depressed with this going on. It d-, uh, it, a couple of days it gets a little discouraging ‘cause you- you just can’t, you want to get out of the house.”* (Participant 411)
	*“Well, it might be what I’m kind of battling or kind of … I feel quite down sometimes, but it … I, I attribute it to the fact I can’t … Frustrating not being able to do what you think you should be able to do.”* (Participant 416)
	FRUSTRATION, ANGER, AND IRRITABILITY
	*“Uh, I don’t know if it’s because of the pandemic, but I feel a little bit more uptight. You know? Um, I, I get angry sometimes a little easier over something stupid, you know. But, but, yeah, I just feel a little bit anxious*.” (Participant 414)
	*“Um, I, I’ve noticed that your, your mood is a little better, right? When you were, when it was cool and, um, in the beginning I, I noticed, you know, I think you were a little more, um, you would probably feel more frustrated and, you know, a little more short and those kinds of things because you were cooped up in the house all the time, right?”* (Participant 414’s Spouse)
	SLEEP
	*“Oh, the only thing I have a little bit more problem sleeping at times, I think, it’s a little tougher to sleep. I’ve found that. More of a broken sleep. If I wake up, it’s hard for me to get back to sleep.”* (Participant 400)
	*“Other than, maybe a little more variable from day to day. Some nights I have, you know, trouble falling asleep. Other nights, it’s, depending on who knows, whether it’s what I’m eating or, you know, some of it’s just variable. Like last night I had real trouble sleeping. And there was really nothing particularly on my mind, it’s just, you know, it’s physiologically, uh, you know, somewhat different from day to day.”* (Participant 410)
	*“Oh yeah. My sleep quality is… it was poor before, but it’s worse now. And it’s scary, and there isn’t anyone out there to help, help me, and if I, if I had a problem, I, I understand completely why people don’t go anywhere near the ER. You don’t want to go (laughs). It’s just really hard to get rested.” (Participant 413)*

#### Pharmacy and Medication Availability

Participants reported some uncertainty around the restrictions put in place and expressed concerns about how insurance works with increased dispensing fees (Table 1; Subtheme 1.2a Pharmacy and Medication Availability: Dispensing Fees). Some participants reported a change in routine for obtaining their medication, including the new delivery service provided by pharmacy or shift in routine (Table 1; Subtheme 1.2b Pharmacy and Medication Availability: Routine Changes).

#### Virtual Medicine: Facilitators and Recommendations

Mixed responses were provided regarding the transition from in-person to virtual clinical appointments, with an overall preference for participants’ in-person visits. While some enjoyed the benefits of virtual appointments, most viewed it as a temporary solution acknowledging some form of care was better than a complete halt of clinical care. Those who preferred virtual appointments reported the benefits of over-the-phone appointments’ convenience and efficiency, saving them the hassle of driving and parking (Table 1; Subtheme 1.3a Virtual Medicine Facilitators). Most PD patients reported a preference of seeing their physician due to various barriers they viewed in virtual appointments including simultaneous speaking making it difficult to converse, less connection without being physically present viewed as invaluable and increased difficulty conveying notes or updates virtually (Table 1; Subtheme 1.3b Virtual Medicine Barriers). When asked what could be improved in health care during this pandemic to provide the optimal quality of care during these circumstances, some patients provided insightful recommendations including secure email and video methods to efficiently and remotely connect with physicians (Table 1; Subtheme 1.3b Virtual Medicine: Recommendations).

#### Absence of Motor Examination

Many patients reported that the appointment’s motor examination was less useful to them and emphasized its importance for their neurologist. Some patients reported a virtual appointment with video could be useful to assess walking, but no significant concerns were expressed with the inability to fully perform a physical examination of motor symptoms during virtual telephone appointments (Table 1; Subtheme 1.4 Absence of Motor Examination).

#### Worsening of Motor Symptoms

Participants described the worsening of motor symptoms of PD since the pandemic started. Some participants had difficulty differentiating if the worsening of their symptoms was attributed to the disease progression or the pandemic. Others explicitly stated noticing an increase in OFF-times, tremors/shaking, and stuttering (Table 1; Subtheme 1.5a Worsening of Motor Symptoms).

#### Worsening of Non-Motor Symptoms

During the pandemic, PD patients expressed an increase in anxiety and stress, leaving some participants noticing more substantial changes as a result (Table 1; Subtheme 1.5b Worsening of Non-Motor Symptoms: Anxiety and Stress). Surprisingly, when asked about any mood changes or changes to mental well-being since COVID-19, most participants expressed little to no observed changes in their mood (Table 1; Subtheme 1.5b Worsening of Non-Motor Symptoms: Mood). Some participants expressed an increase in more negative moods, including frustration, anger, and irritability since the pandemic (Table 1; Subtheme 1.5b Worsening of Non-Motor Symptoms: Frustration, Anger, and Irritability). Lastly, many participants reported worsening of sleep during the pandemic, expressing increased difficulty falling asleep and a broken sleep during the nighttime, and increased frequency of daytime naps (Table 1; Subtheme 1.5b Worsening of Non-Motor Symptoms: Sleep).

### Impacts on Personal Life

#### Behavioral Changes to Exercise

Many participants reported abrupt changes in their lives with both cancellation and alteration of multiple routine social and physical activities they were engaged in. Specifically, physical activities have been predominantly impacted by PD participants. Many participants found ways to continue a modified exercise routine despite the closure of many in-person physical activity classes and facilities. Many participants reported their modified exercise routine during the pandemic now includes more walking outside during pleasant weather and adapting to virtual exercise classes either on their own via YouTube or following the Parkinson Association of Alberta exercise classes. PD patients reported adjusting to exercising following these virtual exercises classes including yoga, gentle PD exercises, seated exercises, and stretching. PD patients continue to find a modified exercise routine as many have found improvement in their PD symptoms from exercise (Table 2; Subtheme 2.1 Exercise Changes).

**Table 2.** Theme: impacts on personal life

Subtheme	Example Quotes
2.1 Behavioral Changes to Exercise	*“I do an exercise program at the [Alberta city] [gym] with a personal trainer. My wife and I do. We’ve kept that on. Well, [gym] is closed as all health clubs are. But she [neurologist] does a routine for me on the… Virtual on the computer, so I can see the exercise she wants me to do. She does it and then my wife and I, we do it in the basement.”* (Participant 406)
	*“Doing exercise program with stretching and stuff. And another thing I probably do miss, is, uh, I, I, attending yoga classes. But, I, uh, you know, I can do yoga on my own, so. They’re, its gone virtual also. Well I, I just do my own stuff.”* (Participant 412)
	*“I found this, uh, program on, uh, on YouTube that, uh, I really like and it’s, it’s, I think it’s, it’s a lot vigorous exercise than, uh- … than the Parkinson’s one. I don’t know if it’s quite as good. Uh, you can follow him. He’s designed very specific for, for Parkinson’s, but, uh, it’s a good workout, and I’ve doing that almost daily.”* (Participant 409)
	*“I’ve been depending on the association, uh, for a lot of support, and we have a month, we actually, it’s this Thursday. And, um, uh, support group meeting. It’s over the phone. We have a teleconference; it’s not on Zoom. That’s been a consistent thing, along with the exercise groups, and I’ve been doing quite a few different ones. Some of them are still available, like the dancing one and some of the exercise classes. Others, uh, you know, are temporarily suspended, I guess. But that’s, yeah, that’s kind of my main engagement.”* (Participant 405)
2.2 Isolation	*“And when I go into town to shop, like [Participant name] hasn’t been off the farm or seen anyone. And so mental health, this is really tough. Like I haven’t been off the farm since, for the last two months since the, the pandemic was announced. And myself, I feel like a caged cat.”* (Participant 421’s Spouse)
	*“One has to deal with this isolation, as I said, because I’m on my own now. It’s a little more challenging. You can only do so many puzzles.”* (Participant 405)
2.3 Managing Boredom	*“I might go for longer bike rides, I guess. I don’t know. I haven’t found my way through that one. Right now we’re spending an awful lot of time doing nothing, so I’d like to change that.”* (Participant 403)
	*“I’ve been doing hobbies around the house that probably should have been doing for some time, but just haven’t had time to do. Repairs and this, that and the other thing, a little more cleaning than I’m used to doing, that sort of thing.”* (Participant 400)
	*“Well I’ve got a whole list of things that I wanna do, (laughs). So, I- I feel that, you know, I’m looking forward to getting into them, um, is, takes away any boredom. Well, I just finished, (laughs), my, um, quilt top for, uh, my last granddaughter… and now I’ve- I’ve got, uh, five to make for my grandsons, (laughs), so. Um, we’ve been doing some organizing in our basement, and- and garage.”* (Participant 408)
2.4 Interpersonal relationships	*“I think the hardest is not being able to, um, uh, have my grandchildren come over and give them a hug. And the same thing with my parents, who are in their eighties. Um, we’ve tried to, y- you know, we- we’ll drive by and wave at them…and stuff and to talk to them a lot on the phone, but not being able to, um, give our loved ones a hug.”* (Participant 417)
	*“So her and her- her partner came over yesterday and we just sat in the driveway and had a visit, but we did a Zoom every day or something. So to handle all… And we did a virtual family things, like we’re doing here now, we just learned to do this. My wife gets her six siblings on the phone in Ontario and, once a week. And we did this, I do this with my siblings, and- and we have, uh, tomorrow we do, some of us have retired from [company] to, uh, a virtual, uh, happy hour e- every couple of weeks, covering people from Nova-, from Newfoundland to BCs, so it’s kind of fun.”* (Participant 411)
	*“We both recognize that being in closer quarters for longer periods of time and not being able to get out as much, it’s going to be a strain on the relationship, but it’s been pretty good so far. I mean, hell, we’ve been married for like 32 years. I think if anything, it’s got a little better. We’ve always had a good relationship, and I think we’re a little more cognizant of giving the other person a break because we know both of us are going to feel a little bit irritated by the things we can’t do.”* (Participant 401)

#### Isolation

While some people live together with family and others alone, most of the participants have expressed missing their usual daily activities outside the home and visiting with friends and family taking a toll on their well-being (Table 2; Subtheme 2.2 Isolation).

#### Managing Boredom

While some participants have expressed feelings of isolation, others are searching or have found new activities to manage the boredom that comes from being isolated in their homes (Table 2; Subtheme 2.3 Managing Boredom).

#### Interpersonal Relationships

Participants frequently reported adhering to the physical distancing measures by eliminating friends and family regular indoor visitations. Many participants expressed being grandparents and found it very difficult to be stripped of visiting their grandchildren during this time. Furthermore, participants expressed ways to keep socializing amid the pandemic by adapting to use technology, phone or video calls, or visiting outdoors with physical distancing measures. However, most of the perceptions around socialization, despite the attempts to connect via technology, were longing to give and receive hugs of family members and be present physically. In terms of spousal relationships, participants shared a mix of reports, with some expressing increased appreciation for their spouse during the pandemic, while others felt the strain of living in close proximity (Table 2; Subtheme 2.4 Interpersonal Relationships).

### Attitudes and Perceptions

#### Silver Linings of COVID-19

While the pandemic has affected many individuals in negative ways, some of the participants expressed the contrary. They saw some positive changes emerge from the new way of life, including thoughts on their PD being more comfortable managing from home and a slower-paced life, more consistency in meals and medications, and saving time and money on driving (Table 3; Subtheme 3.1 Silver Linings of COVID-19).

**Table 3.** Theme: attitudes and perceptions

Subtheme	Example Quotes
3.1 Silver Linings of COVID-19	*“What we’ve liked. Well actually I don’t mind working from home… so I can’t say that’s bad. I (laughs), I, I, I actually don’t mind it. I, you know, it, it’s, I, um, my routine is a little easier in the morning because usually I have to get up and get going and, um, help Interviewee with all of his stuff and get myself ready. So I. I might now look like I usually do when I go to work, but it’s a little less work not to have to do your hair and your makeup and everything to go to work. I said I might ask if I can at least do one or one day at home a week.”* (Participant 414)
	*“I just find that people are just very kind to each other. They seem to care, uh, they seem to check on you. They bring you little treats, those kinds of things that maybe normally wouldn’t happen. And because COVID, in many respects, has brought out the best in some people, and but it, it’s, it’s, so fortunately, if, if we just see the light at the end of tunnel, so I think it, people are a little bit more relaxed and realizing if they come over and they’re not as depressed, but they certainly have been supportive and kind and friendly.”* (Participant 415)
	*“But you know, I’m- I’m putting some things together that I bought, or ready-made meals there, and uh, you know, making some creative adjustments to a frozen pizza. It’s amazing what you can do (laughs). So, uh, so that’s not too bad, and uh, you know, I- I’m a little more consistent now in eating at regular times.”* (Participant 405)
3.2 Attitudes Around Health Experts Recommendations	*“Not really. I’m, I’m aware of precautions that should be taken and, uh, I mostly take them- So I’ve, I’m not fearful. But we are then, uh, uh, we understand why it’s necessary and, and our, and our patient and committed to doing our part.”* (Participant 402)
	*“All we’re doing is following the basic rules and staying home and, uh, going out for groceries sort of, uh, periodically when we need to.”* (Participant 410)
	*“We try to explain that it’s not so much keeping you free of the virus, but if you get it and then pass it along to papa, then that’s a problem. So I’ve been the threat not intentionally, but that’s the way it is. We had to explain to them why it’s really important that they’re judicious about their hygiene.”* (Participant 403)
3.3 Personal and Societal Concerns	*“But that’s my biggest issue, is, uh, the Parkinson’s, um, I think I read somebody passed away in one of these nursing homes recently in, it was either Calgary… I saw it on TV, anyway. And he had Parkinson’s.”* (Participant 411)
	“*Yeah, I’m a little concerned about my income. It’s getting smaller and smaller and unfortunately, prices of everything are going up from taxes to food to, I don’t know what else. So I’ll just have to see how that goes.”* (Participant 407)

#### Adhering to Health Experts Attitudes and Awareness

Many participants reported abiding to public health measures in place and minimizing risks for getting infected. Few patients reported having comorbidities and felt at even greater risk. Besides, when asked about travel and socialization restrictions, most participants understood where the priority lies with the health experts in safety precautions even if they miss their usual activities. Participants’ attitudes were negative around those who do not adhere to public health measures (Table 3; Subtheme 3.2 Attitudes Around Health Experts Recommendations).

#### Personal and Societal Concerns

While many participants expressed minimal fears or concerns about COVID-19 because of the inability to do anything about the situation, others felt different and skeptical of the current situation and what the future will hold, including health, finances, and government decisions (Table 3; Subtheme 3.3 Personal and Societal Concerns).

## Discussion

In this single-center study, initial evidence is provided into the clinical and personal impacts of COVID-19 pandemic on persons living with PD through a qualitative approach. Researchers have highlighted the importance of qualitative research during the COVID-19 pandemic, reporting the value in gaining insights into aspects of behavior and perceptions that is often missed in epidemiological and clinical research.^16^ This study allowed building the interview guide accordingly to ask questions on PD patients lived experiences during COVID-19, clinical care and epidemic response efforts to complement epidemiological data. While these interviews did identify PD patients coping reasonably well with the pandemic changes, adverse consequences arose impacting their quality of life, including lack of access to necessary health care, worsening symptoms, and social isolation for others that were explored in depth. These domains provide insight into modifications and restructuring of treatment planning for the subsequent waves of COVID-19 (at the time of writing Calgary, Alberta is currently experiencing a second wave). A benefit of the study was the in-depth data collection that allowed a rich understanding of the experiences of PD patients in an urban, ambulatory care clinic context that is transferable to other outpatient, community-based clinical settings providing virtual care.

### Medications

The takeaway from our PD participants was more associated with the uncertainty of pharmacy procedures around PD medications more so than the direct impacts. PD patients of our study coped well with adapting their routines with minimal difficulty. Several provinces in Canada, including Alberta, imposed limits on the supply of prescription drugs that pharmacies can dispense. These restrictions took place in March 2020 and placed a 30-day limit of medication instead of the usual 90 or 100 d creating further impacts for PD patients. While PD patients in our study faced slight disturbances in their ordinary routine regarding obtaining their medication and payments, many had questions around increased dispensing fees. In particular, since they normally would receive 90-days’ worth of prescription medication, they were now paying the dispensing fee three times instead of one. As of June 15, 2020, Alberta’s pharmacies have resumed dispensing up to a 100-day supply for most medications. In one study by Cheong and colleagues,^17^ out of 346 participants, 45.4% answered ‘yes’ to difficulty obtaining regular PD medication due to COVID-19, while 54.6% answered ‘no’. These findings support our participant’s experiences as those who did express some effect was minimal. The takeaway from our PD participants was more associated with the uncertainty of pharmacy procedure around PD medications more so than the direct impacts. PD patients of our study coped well with adapting their routines and would have no patients reported difficulty receiving their PD medications.

#### Next steps

Increased communication around pharmacy and medication availability would help in future restrictions of PD medications for individuals. Parkinson Canada, a main information source for PD news, recommends individuals with PD to check their medications and contact pharmacists to refill prescriptions that are running low ahead of time to ensure sufficient time for processing and delivery.^18^ Further, Parkinson Canada recommends taking advantage of delivery service if available to limit exposure in the pharmacy.^18^ Similarly, the Michael J. Fox Foundation for PD research provided recommendations on their website in regard to PD medications during the pandemic with the guidance of a movement disorders specialist.^19^ Some insurance companies are lifting restrictions that typically only allow a one-month supply. PD individuals are encouraged to maintain a 3-month supply of medication and contact their doctor and pharmacist about options if needed. Michael J. Fox Foundation reemphasized taking advantage of delivery service or an insurance mail-order service during these times.^19^ It is important to adhere to these recommendations, as limiting supplies to 30 d can increase the risk of contracting COVID-19 by creating the need to go to public places, including pharmacies, more frequently. We encourage PD individuals to stay informed on updates. Future research and policies should encourage pharmacies to facilitate more open communication about PD medications and insurance plans during changes in medication procedures.

### Clinical Care

Many PD patients routinely visit with their neurologists at the Movement Disorders Clinic for physical motor assessments and medication adjustments. However, only a limited number of patients, such as PD patients receiving Botulinum injections, have returned to in-person visits. Many non-surgical procedures have been postponed preventing patients from potential exposure to the virus. Some elective surgical procedures like deep brain stimulation (DBS) have been delayed, leaving some PD patients facing barriers in achieving optimal care until further notice.^20^ Only one participant in this study was waiting for his DBS appointment, which has been cancelled for the time being, while many other participants expressed a short-term understanding of the delay in clinical care but foresee progressive PD problems if the clinical visitations do not resume soon.

Hospital visits should still be avoided to those who do not have an urgent need to be in person for their clinical appointments whenever possible during this period. Ample research has come forth with telemedicine’s validity to assess PD patients virtually, including visualization of physical examinations by videotaping or video consultations.^1^ While many participants in this study seemed indifferent to the motor examination, assessing rigidity and postural reflex impairment can be more complex for the neurologist to assess. The MDS-Unified Parkinson’s Disease Rating Scale (MDS-UPDRS) used to evaluate various aspects of PD, including motor and non-motor experiences of daily living and motor complications, has been compromised to the lack of physical examination.^21,22^ Due to the inability to perform muscle rigidity and retropulsion pull testing as part of the MDS-UPDRS via videoconference, a modified version has been created that is still reliable and valid, omitting the last pieces.^23^ Other than these two described “hands-on” maneuvers, the remaining examination of the MDS-UPDRS, including gait assessment, can be completed if patients are not at high risk of falls. Other routine testing in PD clinical appointments include the Montreal Cognitive Assessment (MoCA), diagnosis of PD, and atypical parkinsonian syndromes that all seem to be promising to be conducted over virtual means rather than in person.^24-27^ Many participants’ comments regarding virtual medicine align with the results from a recent online survey of 781 PD patients who expressed their main concerns with telemedicine: the lack of hands-on care, lack of intimate and personal interaction with physicians and difficulties in navigating technology.^28^

To continue to support patients and professionals within the PD community regarding ongoing health care, the International Parkinson and Movement Disorder Society^29^ has developed a practical step-by-step guide for implementing telemedicine. Despite some barriers, telemedicine for PD care is feasible. It can even be advantageous in terms of access to physicians, convenience, saved time, and cost reductions.^30^ Telemedicine can be a valuable tool for PD patients who have difficulty travelling to the specialty clinics for their appointments due to distance, mobility, or cognitive impairments.^30^ One study by Qiang and Marras^31^ found virtual appointments saved PD patients an average of 209 min in travel time, 160-km travel distance and $200 (CAN).

This approach aligns with the perceptions of participants of this study who mentioned preference over the quick virtual appointments and avoiding the driving, gas, and time usually spent. In the previously mentioned online survey study, overall satisfaction rates were high, with 97% of patients and 86% of physicians reported being satisfied or very satisfied with the virtual visits.^31^

Despite personal preferences, evidence in the field shows no significant changes in outcome between telemedicine and in-person care.^32-34^ One study comparing virtual clinical care visits via an iPad mini to regular in-person visits every two months reported no significant differences in quality of life measures including Parkinson’s Disease Questionnaire-39, UPDRS parts I and II, motor scores (UPDRS parts III and IV, Modified Hoehn and Yahr stage), mental health measures (Beck Depression Inventory), number of phone calls, or number of hospital visits.^34^

#### Next Steps

To better address both motor and non-motor symptoms of PD a virtual care policy in Ontario Canada provides useful recommendations for remote clinical care.^35^ There has been a lack of long-term policy developments for virtual care. This study asked patients recommendations and opinions on future clinical care and results reported that prospective studies continue to promote patient centered outcomes and acknowledged the focus on patient’s needs during virtual care. Meaningful engagement and discussions could help understand how and why patients would use virtual care technologies. Adopting a similar approach would target a more system-wide investment in Canadian virtual care implementation strategies.^35^ Specifically, findings of the consensus policy dialogue indicated that patients recommended communication with their primary health care providers to be reciprocated within 24 h regardless of virtual method. This finding suggests that patients may not provide indicators directly about technology methods of practice but rather meaningful patient engagement in health care policy planning of virtual care could be promising future direction for research.^35^ This study aligns with patient-centered primary and community care needed to show importance for two key implications for more implementation of virtual care in clinical care. Qualitative work presented here provides the base for building up the understanding of the best way to measure the effectiveness and quality of virtual care after such a rapid shift to this modality of care provision.

### Motor Symptoms

Some participants noticed worsening of their PD symptoms, including increased OFF-times, tremors/shaking, and stuttering (see Table 1; Subtheme 1.5a Worsening of Motor Symptoms). The increased psychological stress during the COVID-19 pandemic can temporarily worsen various motor symptoms, including tremor, freezing of gait, or dyskinesias.^36,37^ Psychological stress may reduce the efficacy of dopaminergic medication, which we hypothesize may explain the reported prolongation of OFF-period duration.^36^ Existing evidence supports that the pandemic induced a significant worsening of motor performance and motor-related disability. Further, the levodopa medication response worsened, and patients saw an increased daily OFF-time, caused by either acute systemic inflammatory response or changes in pharmacokinetics.^37-41^ A study by Song et al.^42^ collected data on prescribed medications for both motor and non-motor symptoms of PD and found no significant changes of symptoms causing medication adjustment between two clinic visits during the pandemic.

We also discussed changes in patients’ exercise routines. We believe that a decrease in patients’ mobility from their usual physical activities has worsened their PD, including other therapies such as physiotherapy. A significant association between reduced exercise amount and subjective worsening or both motor and non-motor symptoms of PD was reported despite no significant change in the UPDRS part 3 scores.^42^ In a study by Zipprich et al.,^3^ 31.3% of PD patients complained about decreased mobility supporting our findings. Despite the decrease in activities, many participants in this study noted the following virtual exercise programs or spending a lot more time walking outdoors. This aligns with the findings in Zipprich et al.’s study in which 34.3% of the participants with PD had to refrain from sports activities.^3^ Of the total, 82.4% found alternative means to continue exercises at home or to go outside.^3^ It is important to note that participants’ comments occurred as the weather transitioned into the spring season. We anticipate a decrease in outdoor activities and walking during the upcoming winter months if restrictions are still in place for PD patients and anticipate an increase in feelings of isolation and confinement.

### Nonmotor Symptoms

We discussed the worsening of non-motor symptoms for PD patients during the pandemic. Previous studies also reported that experiencing anxiety and worrying about the current situation were reported by 58.6% of patients.^3^ Worries and fears revolved around contracting the virus and being at risk due to their PD or other comorbidities, or fears of loved ones contracting it. Two of the apparent non-motor symptoms in this study were decreased sleep quality and an increase in fatigue. A recent study by Xia et al. investigated how the pandemic has affected sleep and mental health of patients with PD in comparison to healthy controls.^43^ Results of their study showed that 68.9% of the PD patients suffered from sleep disturbance, a much higher rate than that of the general population. Further evidence shows that scores from the Hamilton Anxiety and Depression Scale on PD patients with sleep disturbances were significantly higher than PD patients who did not experience sleep disturbances, indicating that anxiety and depression are important factors affecting PD patients’ sleep quality during the pandemic.^43^

#### Next Steps

For monitoring both motor and non-motor symptoms of the disease during the pandemic have been explored in a recent study by Miele et al. who investigated teleconsultations for overcoming outpatient clinic restrictions.^44^ They recommend an easy to fill electronic diary may be useful to track daily activities, symptoms of motor and non-motor, feelings and general health. The electronic diary is available for both iOS and Android platforms and is called “The Parkinson’s diary” on the app store.^44^ This new strategy is based on similar smartphone application research with a strong correlation in the MDS-UPDRS-III score that shows promising results for evaluating clinical signs that otherwise could not be collected virtually. As our results show that PD patients with both motor and non-motor symptoms have been impacted during the pandemic in varying ways therefore, we would support the recommendation by Miele and colleagues for PD patients to monitor physical and mental status during the pandemic.

### Challenges and Future

We identified some challenges reported by PD participants, including struggles with changes in an exercise routine, feelings of isolation and boredom, and personal concerns about what the new normal will be moving forward. In the study by Zipprich et al.,^3^ 72.7% of participants reported changes in behaviors since COVID-19 emerged. Their results are aligned with our findings that participants’ fears and concerns are that the virus is dangerous in general or to them personally and see themselves as patients of risk.^3^ Isolation and limited visitation with family and friends have been common responses expressed by participants. Other challenges include uncertainty about economic and social developments for the future and what clinical care will look like if this continues. On the other hand, we found a handful of participants who took a positive stance on the impacts of COVID-19, including statements on enjoying a slower-paced life. Future research should be conducted to examine if there are positive impacts on the pandemic’s behavioral changes as most of the existing evidence has focused on the concerning impact and effects. Prospective research should also consider more patient engagement in virtual care policy and implementation.

### Limitations

The present study has several limitations. This study used a pre-selected sample of patients who have shown interest in research or have had possibly more research exposure than the general PD population of the clinic. Another limitation of the study is selection bias favoring this pre-selected sample that perhaps were relatively healthier and had readily accessible technology including phone, internet, email. We aimed at accommodating for less accessible technology by incorporating oral consent via phone for those patients who were unable to access internet and computer. Due to the restrictions and suspension of in-person clinical visits, we were not able to perform any clinical assessments such as MDS-UPDRS at the time; however, it is worth looking into the use of the virtual adaptations to assessments as noted in the study for future studies where in-person assessment is not possible. Future work should aim to incorporate both the perspectives of patients with PD exploring more symptoms and managing the disease. While our study was inclusive of caregivers/spouses of participants if they wished to join, our main interest was at the lived experiences of those with the PD diagnosis therefore we did not have enough caregivers attend or data to generate unique themes across caregivers for this study. A prospective study could be useful in exploring the caregivers lived experiences during the pandemic.

## Conclusion

In conclusion, this study provided an overview of PD participants’ experiences during COVID-19 with an emphasis on clinical aspects and remote care. Three major themes PD patients reported as a result of the pandemic were impacts on clinical care including, personal life, and changes in attitudes and perceptions. The most significant theme was how the pandemic affected PD clinical care including health care and medication limitations, how the transition to virtual care affected PD patients and what is needed moving forward to improve telehealth and symptom management. Suggestions from our participants include increased communication with specialists around clinical care and recommendations for continued clinical management of symptoms amid the pandemic. Overall, this study provides a unique opportunity for researchers to better understand the lived experiences of PD patients in all aspects of their life suggesting innovative means are needed for facilitating virtual health care medicine and increased social interaction to help mitigate future pandemics or anticipated futures waves of COVID-19, extreme weather phenomena or other similar emergencies.

## Supplementary material

For supplementary material accompanying this paper visit http://dx.doi.org/10.1017/cjn.2021.7. click here to view supplementary material
