# Supplementary material for: Effects of the COVID-19 Pandemic on Parkinson’s Disease: a Single-Centered Qualitative Study
Source: Can J Neurol Sci. 2021 Apr 12:1–13. doi: 10.1017/cjn.2021.70 (PMC8160494; doi:10.1017/cjn.2021.70)
Supplement: Supplementary file 1 [file S0317167121000706sup001.pdf]

Hello, I'm [interviewer's name]. Thank you for agreeing to talk to me today.

We've invited you here today because you usually go to [insert clinic name].

We know being socially connected and able to receive all the services you are used to is very important to live as well as possible with any neurological or psychiatric condition. We also know that with the emergence of coronavirus [COVID-19 (SARS-CoV-2)] as a new virus for Canada this year there may have been many changes in your usual routine and services. In mid-March 2020 the province of Alberta implemented social distancing measures and placed limits on the number of people permitted in public social gatherings. By the end of March 2020 further public health measures were put in place that limited the provision of community-based programming, and non-emergency/non-urgent clinical care.

These measures have impacted patients and families in many ways, causing disruption in routine, increased stress in trying to continue managing essential needs such as groceries and medications and doing so without the typical social and health service support, or support from personal networks. We also know that many people have now started to have appointments with their doctor on the computer or phone. It is important for us to understand how the social distancing and isolation measures are impacting you and the changes you are experiencing so we can learn better ways to help you and to improve this virtual care.

I want to assure you that everything you share with us today will be kept confidential. The recording of today's session will be transcribed and will undergo further analysis by the research team, and any identifying information will be removed. Only members of the research team will have access to this information.

I expect this interview to last between 30 and 60 minutes. If, at any point you need to take a break or cannot continue, please let me know and we can take a break or reschedule, or stop all together.

Do you have any questions before we begin? [Answer questions as needed]

<u>To start, we have a few questions relating to the pandemic itself:</u>

Have you been diagnosed with Covid-19: [ ] yes [ ] no [ ] not sure

Have you been tested for Covid-19: [ ] yes [ ] no [ ] not sure

If you haven't been tested, do you think you have had any symptoms that might have been Covid-19:
[ ] yes [ ] no [ ] not sure

Has anyone in your household been diagnosed with Covid-19: [ ] yes [ ] no [ ] not sure

At any time, have you been told by a public health authority that you should isolate yourself because of Covid-19 risk: [ ] Yes [ ] No [ ] Not sure

1. Tell me about the kind of help you or your family member(s) had before the coronavirus pandemic? [pre-March 2020]
   Changes regarding:
   - Family caregivers
   - Private care
   - Home care

2. Tell me about the kinds of social activities or day programs you or your family member(s) had before the coronavirus pandemic? [pre-March 2020]
   Changes in terms of:
   - Parkinson groups activities
   - Day Programs

3. Tell me about the types of changes that have been hardest for you or your family since the pandemic started?
   a. Changes to treatment/diagnostics/testing…
      Do you feel that the cancellation of in person appointments with your neurologists has affected the quality of your care?
   b. Probe around fear of seeing the doctor / avoiding appointments vs fear of coronavirus
   c. Boredom, diet/food, social media/news media, physical activity
   - Did you notice any increase in your Parkinson related anxiety?
   - Did you notice any increase in your Parkinson related depression?
   - Are you experiencing any increase in the frequency of perception disorders/hallucinations?
   - Are you noticing any worsening in your ability to control our impulses while you are at home? (such as an increase tendency to consume social media, gamble online, excessive sexual thoughts? excessive eating or shopping online)
   - Did you notice any worsening in your sleep quality?
   - Do you feel interested in pursuing new recreational activities at home now that you can not follow your routine during the pandemic? [Yes, no, why]

4. What kinds of things have you or your family done to help cope with the changes, positive or negative?
   a. Other kinds of formal help
   b. Self-management / physical activity
   c. Alcohol, food, financial concerns (online shopping or gambling)
      Do you feel that the relationship with your partner/caregiver has changed since the beginning of the COVID19 crisis? [Yes/no/why]
5. Can you tell me about changes that might be helpful or that you like since the pandemic started?
   a. Family time
   b. Connectedness/pets

6. Tell me about how ‘virtual’ doctor appointments are going for you or your family member(s)?
   a. Worries/fears

b. Do you feel that the phone follow ups are useful for you?
c. Do you have any particular fear/worry related to your PD?

Example Prompts:

- Medication changes/availability
- Unpredictability of motor symptoms
- Non motor symptoms management (give examples)
- Relationships in the household
- Caregiver burden
- Lack of immediate access to your neurologist/nurse
- Inability to continue your physical exercise routine
- Delay in the access to more invasive treatments

d. Do you have any particular fear related to the effects of COVID19 on Parkinson disease?
e. What would make it better

7. What kinds of things would be helpful for you during social distancing?
   a. Health care
   b. Virtual consults using video instead of phone?
   c. Social care/social engagement

   Parkinson Association of Alberta virtual meetings? Groups?

Now that we are wrapping up our interview, I would like to take a moment and summarize the key points we have covered. (a,b,c...)

[Thank the participant(s) and let them know that if they think of anything else they would like to add, they can contact you]
